# Supplementary material for: Translational Strategies to Target Metastatic Bone Disease
Source: Cells. 2022 Apr 12;11(8):1309. doi: 10.3390/cells11081309 (PMC9030480; doi:10.3390/cells11081309)
Supplement: Supplementary file 1 [file cells-11-01309-s001.zip › cells-1582863-supplementary.pdf]

Table S1. Therapeutic Modalities to Treat Metastatic Bone Disease

Conventional Therapies			
**Modality**	**Examples**	**Effect on Bone**	**Possible Off-Target Effects**
Surgical Interventions	Tumor Debulking<br>Arthroplasty<br>Limb Amputation<br>Spinal Decompression	Bone Malformations<br>Limb Removal<br>Infection/Osteomyelitis<br>Prosthesis Complications	Impaired Mobility<br>Thrombosis<br>Dermal Infection<br>Nerve Damage<br>Bleeding
Analgesic	Paracetamol (Acetaminophen)	Palliative Care for Bone Pain	Nausea<br>Liver Damage<br>Fatigue<br>Paleness<br>Diaphoresis
NSAIDs	Ibuoprofen<br>Naproxin<br>Celecoxib	Palliative Care for Bone Pain	Nausea<br>Liver Damage<br>Fatigue<br>Hemorrhage<br>Anemia<br>Hypertension/Stroke
Corticosteroids	Dexamethasone<br>Prednisolone<br>Hydrocortisone	Palliative Care for Bone Pain<br>Increase Bone Resorption<br>Reduced Osteoblast and Osteocyte Function and Number<br>Osteonecrosis<br>Growth Deceleration (in Children)	Weight Gain/Cushingoid Features<br>Irritability<br>Suppression of Hormone Production via Hypothalamic-Pituitary Axis (Adrenal Atrophy)<br>Neuropsychiatric/Depression<br>Gastrointestinal Complications<br>Liver Damage<br>Cardiovascular Effects<br>Skin Alterations<br>Cataracts
Opiates	Tramadol<br>Codeine<br>Buprenorphine	Palliative Care for Bone Pain<br>Increase Fall-Risk<br>Induce Bone Loss	Addiction<br>Nausea<br>Constipation<br>Vomiting<br>Drowsiness
Chemotherapeutic Agents	Anti-Metabolites<br>Taxanes<br>Topoisomerases<br>Proteasome Inhibitors	Impairment of Kidney Function<br>Block Osteoblast Maturation<br>Increase Bone Resorption	Anemia<br>Fatigue<br>Impaired Cognition<br>Muscle Weakness
Hormone Deprivation	SERMs<br>Aromatase Inhibitors<br>Luteinizing Hormone<br>Enzalutamide	Increase Osteoclast Activity<br>Increase Bone Resorption<br>Inhibit Androgen and Estrogen Synthesis<br>Osteoporosis	Premature Menopause<br>Muscle Weakness<br>Fatigue
Anti-Resorptive Agents	Bisphosphonates<br>Denosumab (α-RANKL)	Reduce Bone Turnover<br>Block Osteoclast Activity<br>Induce Osteoclast Apoptosis<br>Reduce Bone Resorption	Prolonged Use Can lead to Osteonecrosis<br>Prohibited Use in Adolescents<br>Mild Anti-Tumor Effect in Bone<br>Rebound Bone Resorption and Tumor Progression (following Denosumab holiday)

Anabolic Agents	*Parathyroid Hormone (PTH)<br>Romosozumab (α-Sclerostin)	Induce Osteogenic Bone Growth<br>Decrease Bone Resorption	Reduce Tumor Growth<br>Stiffness<br>Joint Pain<br>Shortness of Breath<br>Muscle Spasm<br>***Previous Black Box Warning Removed (PTH)**
Hyperthermia	n / a	Palliative Care for Bone Pain<br>Inhibit Bone Destruction<br>Induce Bone Formation	Thermal Damage, Blisters<br>Discomfort<br>Blood Clot<br>Bleeding<br>Bone Marrow Ablation<br>Temporary Jaundice

Innovative Therapeutic Strategies

Modality	Examples	Effect on Bone	Possible Off-Target Effects
Small Molecule Inhibitors	TGF-β Inhibitors	Reduce Bone Resorption	Increase Cardiovascular Toxicity
Immunotherapeutic Agents	PD-1 / PD-L1<br>CTLA-4	Unknown	Secondary Inflammatory Response
Senescence	Senolytic Compounds	Reduce Bone Loss	Gastrointestinal Discomfort<br>Shortness of Breath
Cryoablation	n/a	Palliative Care for Bone Pain<br>Secondary Fracture<br>Focal Loss of Bone	Thermal Damage to Adjacent Tissue<br>Bone Marrow Damage
Radionuclide	Radium^{223}<br>Samarium^{153}<br>Strontium^{89}	Palliative Care for Bone Pain<br>Destroy Progenitor Cells in Marrow	Anemia<br>Risk of Infection<br>Bruising
Radiofrequency Ablation	n / a	Palliative Care for Bone Pain	Thermal Damage to Adjacent Tissue from Probe
Palliative Pharmacologics	Corticosteroids<br>Cannabinoids<br>Tricyclic Anti-Depressants	Palliative Care for Bone Pain<br>Increase Bone Resorption (Corticosteroids and Tricyclic Anti-Depressants)	Unknown

Table S2. Highly Specific Therapies to Target Metastatic Bone Disease (Not Discussed in the Main Text)

Therapeutic Target	Examples	References
Wingless Integrated (Wnt)	Wnt3a<br>Wnt5a	• [247-250]
Dickkopf-1 (DKK-1)	BHQ880	• [251]
NOTCH	γ-Secretase Inhibitor	• [252-253]
Mammalian Target of Rapamycin (mTOR)	Everolimus	• [254-255]
Tyrosine Kinase Inhibitors (TKI) of:<br>Epidermal Growth Factor Receptor (EGFR)<br>Human Epidermal Growth Factor Receptor Type 1,2 (HER1,2)<br>Rearranged During Transfection (RET)<br>Mesenchymal Epithelial Transition (MET)<br>Vascular Endothelial Growth Factor-A (VEGF-A)<br>Breakpoint Cluster Region (BCR)<br>Ableson Murine Leukemia Virus (ABL)	Kinase Inhibitor Target<br>Gefitinib - (EGFR)<br>Lapatinib - (HER2/ERBB2) & (HER1)<br>Cabozantinib - (RET) (cMET) (VEGFR2)<br>Bevacizumab - (VEGF-A)<br>Imatinib - (BCR-ABL)	• [256-262]
CD20 (B Cell)	Rituximab	• [263]

Epidermal Growth Factor Receptor (EGFR)	Trastuzumab<br>Pertuzumab	• [264-266]
Nanomedicine	Caelyx (Doxorubicin in Liposome-Carrier)<br>DepoVax<br>SGT53<br>NK-105	• [267-268]

**Additional References for Treatment of Metastatic Bone Disease**
• [269-277]

References

247. Zhang, J.; Tu, Q.; Bonewald, L.F.; He, X.; Stein, G.; Lian, J.; Chen, J. Effects of miR-335-5p in modulating osteogenic differ-entiation by specifically downregulating Wnt antagonist DKK1. *J. Bone Miner. Res.* **2011**, *26*, 1953–1963.
248. Janda, C.Y.; Dang, L.T.; You, C.; Chang, J.; de Lau, W.; Zhong, Z.A.; Yan, K.S.; Marecic, O.; Siepe, D.; Li, X.; et al. Surrogate Wnt agonists that phenocopy canonical Wnt and beta-catenin signalling. *Nature*, **2017**, *545*, 234–237.
249. Sottnik, J.L.; Hall, C.L.; Zhang, J.; Keller, E.T. Wnt and Wnt inhibitors in bone metastasis. *BoneKEy Rep.* **2012**, *1*, 101, doi:10.1038/bonekey.2012.101.
250. Hall, C.L.; Kang, S.; MacDougald, O.; Keller, E.T. Role of wnts in prostate cancer bone metastases. *J. Cell. Biochem.* **2006**, *97*, 661–672, doi:10.1002/jcb.20735.
251. Fulciniti, M.; Tassone, P.; Hideshima, T.; Vallet, S.; Nanjappa, P.; Ettenberg, S.A.; Shen, Z.; Patel, N.; Tai, Y.-T.; Chauhan,D.; et al. Anti-DKK1 mAb (BHQ880) as a potential therapeutic agent for multiple myeloma. *Blood* **2009**, *114*, 371–379, doi:10.1182/blood-2008-11-191577.
252. Rizzo, P.; Osipo, C.; Foreman, K.; Golde, T.; Osborne, B.; Miele, L. Rational targeting of Notch signaling in cancer. *Oncogene* **2008**, *27*, 5124–5131, doi:10.1038/onc.2008.226.
253. Osipo, C.; Zlobin, A.; Olsauskas-Kuprys, R. Gamma secretase inhibitors of Notch signaling. *OncoTargets Ther.* **2013**, *6*, 943–955, doi:10.2147/OTT.S33766.
254. Hadji, P.; Coleman, R.; Gnant, M. Bone effects of mammalian target of rapamycin (mTOR) inhibition with everolimus. *Crit. Rev. Oncol.* **2013**, *87*, 101–111, doi:10.1016/j.critrevonc.2013.05.015.
255. Baselga, J.; Campone, M.; Piccart, M.; Burris, H.A.III.; Rugo, H.S.; Sahmoud, T.; Noguchi, S.; Gnant, M.; Pritchard, K.I.; Lebrun, F.; et al. Everolimus in Postmenopausal Hormone-Receptor–Positive Advanced Breast Cancer. *N. Engl. J. Med.* **2012**, *366*, 520–529, doi:10.1056/nejmoa1109653.
256. Vandyke, K.; Fitter, S.; Dewar, A.L.; Hughes, T.P.; Zannettino, A.C.W. Dysregulation of bone remodeling by imatinib mesylate. *Blood* **2010**, *115*, 766–774, doi:10.1182/blood-2009-08-237404.
257. Geyer, C.E.; Forster, J.; Lindquist, D.; Chan, S.; Romieu, C.G.; Pienkowski, T.; Jagiello-Gruszfeld, A.; Crown, J.; Chan, A.; Kaufman, B.; et al. Lapatinib plus Capecitabine for HER2-Positive Advanced Breast Cancer. *N. Engl. J. Med.* **2006**, *355*, 2733–2743, doi:10.1056/nejmoa064320.
258. Lu, X.; Kang, Y. Epidermal growth factor signalling and bone metastasis. *Br. J. Cancer* **2010**, *102*, 457–461.
259. Escudier, B.; Powles, T.; Motzer, R.J.; Olencki, T.; Frontera, O.A.; Oudard, S.; Rolland, F.; Tomczak, P.; Castellano, D.; Appleman, L.J.; et al. Cabozantinib, a New Standard of Care for Patients With Advanced Renal Cell Carcinoma and Bone Metastases? Subgroup Analysis of the METEOR Trial. *J. Clin. Oncol.* **2018**, *36*, 765–772, doi:10.1200/jco.2017.74.7352.
260. Donskov, F.; Motzer, R.J.; Voog, E.; Hovey, E.; Grüllich, C.; Nott, L.M.; Cuff, K.; Gil, T.; Jensen, N.V.; Chevreau, C.; et al. Outcomes based on age in the phase III METEOR trial of cabozantinib versus everolimus in patients with advanced renal cell carcinoma. *Eur. J. Cancer* **2020**, *126*, 1–10, doi:10.1016/j.ejca.2019.10.032.
261. Ellis, L.M. Mechanisms of Action of Bevacizumab as a Component of Therapy for Metastatic Colorectal Cancer. *Semin. Oncol.* **2006**, *33*, S1–S7, doi:10.1053/j.seminoncol.2006.08.002.
262. Miller, K.; Wang, M.; Gralow, J.; Dickler, M.; Cobleigh, M.; Perez, E.A.; Shenkier, T.; Cella, D.; Davidson, N.E. Paclitaxel plus Bevacizumab versus Paclitaxel Alone for Metastatic Breast Cancer. *N. Engl. J. Med.* **2007**, *357*, 2666–2676, doi:10.1056/nejmoa072113.
263. Bindal, P.; Jalil, S.A.; Holle, L.M.; Clement, J.M. Potential role of rituximab in metastatic castrate-resistant prostate cancer. *J. Oncol. Pharm. Pract.* **2018**, *25*, 1509–1511, doi:10.1177/1078155218790338.
264. Hynes, N.E.; Lane, H.A. ERBB receptors and cancer: The complexity of targeted inhibitors. *Nat. Rev. Cancer* **2005**, *5*, 341–354.

265. Swain, S.M.; Miles, D.; Kim, S.-B.; Im, Y.-H.; Im, S.-A.; Semiglazov, V.; Ciruelos, E.; Schneeweiss, A.; Loi, S.; Monturus, E.; et al. Pertuzumab, trastuzumab, and docetaxel for HER2-positive metastatic breast cancer (CLEOPATRA): End-of-study results from a double-blind, randomised, placebo-controlled, phase 3 study. *Lancet Oncol.* **2020**, *21*, 519–530, doi:10.1016/s1470-2045(19)30863-0.
266. Tabernero, J.; Hoff, P.M.; Shen, L.; Ohtsu, A.; Shah, M.A.; Cheng, K.; Song, C.; Wu, H.; Eng-Wong, J.; Kim, K.; et al. Pertuzumab plus trastuzumab and chemotherapy for HER2-positive metastatic gastric or gastro-oesophageal junction cancer (JACOB): Final analysis of a double-blind, randomised, placebo-controlled phase 3 study. *Lancet Oncol.* **2018**, *19*, 1372–1384, doi:10.1016/s1470-2045(18)30481-9.
267. Verma, S.; Dent, S.; Chow, B.J.; Rayson, D.; Safra, T. Metastatic breast cancer: The role of pegylated liposomal doxorubicin after conventional anthracyclines. *Cancer Treat. Rev.* **2008**, *34*, 391–406, doi:10.1016/j.ctrv.2008.01.008.
268. Adjei, I.M.; Temples, M.N.; Brown, S.B.; Sharma, B. Targeted Nanomedicine to Treat Bone Metastasis. *Pharmaceutics* **2018**, *10*, 205, doi:10.3390/pharmaceutics10040205.
269. Coleman, R. Metastatic bone disease: Clinical features, pathophysiology and treatment strategies. *Cancer Treat. Rev.* **2001**, *27*, 165–176, doi:10.1053/ctrv.2000.0210.
270. Yasir, M.; Goyal, A.; Sonthalia, S. *Corticosteroid Adverse Effects*; StatPearls Publishing: Treasure Island, CA, USA, 2022.
271. Hall, C.L.; Keller, E.T. The role of Wnts in bone metastases. *Cancer Metastasis Rev.* **2006**, *25*, 551–558, doi:10.1007/s10555-006-9022-2.
272. Suva, L.J.; Washam, C.; Nicholas, R.W.; Griffin, R.J. Bone metastasis: Mechanisms and therapeutic opportunities. *Nat. Rev. Endocrinol.* **2011**, *7*, 208–218, doi:10.1038/nrendo.2010.227.
273. Clézardin, P.; Coleman, R.; Puppo, M.; Ottewell, P.; Bonnelye, E.; Paycha, F.; Confavreux, C.B.; Holen, I. Bone metastasis: Mechanisms, therapies, and biomarkers. *Physiol. Rev.* **2021**, *101*, 797–855, doi:10.1152/physrev.00012.2019.
274. Litwin, M.S.; Tan, H.J. The Diagnosis and Treatment of Prostate Cancer: A Review. *JAMA* **2017**, *317*, 2532–2542.
275. Marcove, R.C.; Miller, T.R. Treatment of primary and metastatic bone tumors by cryosurgery. *J. Am. Med. Assoc.* **1969**, *207*, 1890–4.
276. Santini, D.; Galluzzo, S.; Zoccoli, A.; Pantano, F.; Fratto, M.; Vincenzi, B.; Lombardi, L.; Gucciardino, C.; Silvestris, N.; Riva, E.; et al. New molecular targets in bone metastases. *Cancer Treat. Rev.* **2010**, *36*, S6–S10, doi:10.1016/s0305-7372(10)70013-x.
277. Ono, M.; Kuwano, M. Molecular Mechanisms of Epidermal Growth Factor Receptor (EGFR) Activation and Response to Gefitinib and Other EGFR-Targeting Drugs. *Clin. Cancer Res.* **2006**, *12*, 7242–7251, doi:10.1158/1078-0432.ccr-06-0646.
